# The longitudinal association between change in physical activity, weight, and health-related quality of life: Results from the population-based KORA S4/F4/FF4 cohort study

**DOI:** 10.1371/journal.pone.0185205

**Published:** 2017-09-27

**Authors:** Matthias Rabel, Christa Meisinger, Annette Peters, Rolf Holle, Michael Laxy

**Affiliations:** 1 Department of Sport and Health Sciences, Technical University Munich, Munich, Germany; 2 Institute for Medical Information Processing, Biometrics and Epidemiology (IBE), Ludwig-Maximilians-University Munich, Munich, Germany; 3 Institute of Health Economics and Health Care Management, Helmholtz Zentrum München–German Research Center for Environmental Health (GmbH), Neuherberg, Germany; 4 Institute of Epidemiology II, Helmholtz Zentrum München–German Research Center for Environmental Health (GmbH), Neuherberg, Germany; Seoul National University, REPUBLIC OF KOREA

## Abstract

**Introduction:**

Longitudinal evidence on the association between physical activity (PA) or weight and health-related quality of life (HRQL) is sparse and studies describe inconclusive results. The aim of this study was to examine longitudinal associations between change in PA and HRQL as between change in weight and HRQL respectively.

**Methods:**

Analyses are based on data from the KORA S4 cohort study (1999–2001; n = 4,261, mean age 49.0 ± 13.3 years) and the two follow-up examinations (F4: 2006–2008; FF4: 2013–2014). Information on PA was collected in standardized interviews. Weight was measured objectively. Mental and physical components of HRQL were assessed via the SF-12 questionnaire. First, change in HRQL was regressed on change in PA and weight. Second, hierarchical linear models were fitted, which allowed estimation of between-subject and within-subject effects. Analyses were adjusted for the covariates sex, baseline diseases, and education.

**Results:**

A change to a physically more active lifestyle is positively associated with physical and mental HRQL. Although weight gain is associated with impairments in physical HRQL, the data show an inverse relationship between weight gain and mental HRQL. The results were consistent for both the change score analyses and the hierarchical linear models.

**Discussion:**

Our findings stress the importance of interventions on PA/weight. Nonetheless, more research is needed to reveal the causal relationship between PA/weight and HRQL.

## Introduction

Physical inactivity and overweight impose a huge burden on both individuals and society. The World Health Organization has identified physical inactivity as one of the top global risks of death (6% of global deaths) [1]. Overweight and obesity are in second place with almost 5% of global deaths. With increasing numbers of overweight and obese people, the risk of suffering from coronary heart disease, ischemic stroke, and type 2 diabetes grows steadily [2]. In addition to that, physical inactivity and overweight are linked to substantial higher costs for the healthcare system [3–5].

Health-related quality of life (HRQL) has been described as a crucial parameter for health and health-promoting interventions. HRQL is an important patient-centered outcome. It is an essential concept that is relevant for clinical appraisal. Moreover, valid and reliable estimates of the effect of weight or physical activity (PA) on HRQL are important parameters for decision analytic models [6]. HRQL is described as a multi-dimensional concept that includes physical and mental domains [7]. There are several generic instruments to measure HRQL such as the SF-36, SF-12, EQ-5D, or WHOQOL instruments.

Obesity is inextricably linked to PA [8, 9]. However, although this link has been established, both obesity and PA need to be seen as independent contributors to HRQL.

In cross-sectional study designs, a positive association between PA and HRQL is well documented [10–13]. In addition, the cross-sectional relationship between weight status and HRQL has been examined extensively, with the result of lower HRQL among obese and overweight people [14–17]. Additionally, there is substantial evidence for impaired HRQL among underweight persons. Thus, the cross-sectional relation between the body mass index (BMI) and HRQL can be described as an inverse U-shaped relationship [18–21]. However, cross-sectional studies are often susceptible to potentially biased estimates, and the empirical evidence on the longitudinal association is much sparser.

According to the literature, there seems to be a positive longitudinal association between change in PA and HRQL. Numerous studies present results that indicate a positive relationship between increased PA and HRQL [10–12, 22, 23]. Tessier et al. observed stronger relations between PA change and mental components than physical components of health [22]. A few studies report gender differences regarding the association between PA change and HRQL [12, 22]. Although they observed hints of a positive relation between PA change and HRQL in their systematic review, Bize et al. call for caution in interpreting these results because of methodological limitations [11].

The empirical evidence on the longitudinal relationship between weight change and HRQL is inconclusive. Several studies report impairments in physical HRQL linked to weight gain [14–16, 21, 24–26]. However, some studies report a positive association between weight gain and mental components of HRQL [21, 25, 26]. Döring et al. could not confirm this association. They report an inverse relationship between weight gain and physical HRQL, but none or only weak associations with mental dimensions [14]. Although the relation between weight change and HRQL has gained increasing scientific attention over recent years, the results are still inconclusive [15]. On account of different methodological approaches and analyses, various lengths of study periods, and different study populations, the evidence on this subject is still heterogeneous.

In the present study, we present data from a population-based cohort including three measurement points over 14 years with the aim of further strengthening knowledge of the associations between change in PA/weight and HRQL in the general population. Because of the strong link between PA and weight, this article covers the association between PA and HRQL and between weight and HRQL as separate questions of interest.

## Methods

### Study design and study population

Data from the KORA (Cooperative Health Research in the Region of Augsburg) S4/F4/FF4 cohort study were used. In the course of KORA, a cross-sectional health survey S4 (1999–2001) was conducted with 4,261 participants aged from 25 to 74 years [27]. Since S4, there have been two follow-up examinations: F4 (2006–2008) with 3,080 and FF4 (2013–2014) with 2,279 participants. The loss of participants from S4 to FF4 was the result of participants’ death (455), relocation (296), refusal (570), illness/lack of time (504), and no contact possible (157). In all three studies, participants were physically examined and additional information such as socio-demographics were obtained via standardized interviews. Details about the setting, recruitment, and data collection are described elsewhere [28]. All study participants’ gave written informed consent and the study was approved by the Ethics Committee of the Bavarian Medical Association. The present study is a continuation of the work of Laxy et al. with the addition of a further measurement point and an additional focus on the association between PA and HRQL [25].

### Measures

#### Health-related quality of life

HRQL was measured using the SF-12 health survey, which is a compact version of the SF-36 health survey. It consists of 12 items, six items addressing physical health components and six items representing mental health components of HRQL. The items score eight domains of health and can be summarized to a physical component score (PCS) and a mental component score (MCS) with higher scores indicating better HRQL. Test–retest reliability of the PCS is 0.89 and 0.77 for the MCS in a US population. Median validity for the PCS is 0.67 and 0.97 for the MCS [29].All psychometric quality criteria can be found in Ware et al, 1996 [29]. According to systematic reviews on the relationship between PA and HRQL, the SF-36 and its short form are the most often applied questionnaires for examining the impact of weight or PA on HRQL [10, 11].

#### Weight

Information on weight and height were assessed by anthropometric measurements carried out by trained medical staff. Participants were weighed in light clothes and shoes. Height and weight were measured non-digitally and digitally (SECA 221, SECA 709) allowing accurate measurement up to 0.1 cm or 0.1 kg. BMI was calculated based on weight and height and classified according to the World Health Organization thresholds [2].

#### Physical activity

PA was assessed during the standardized interviews. Participants were asked to report their time per week spent on leisure-time PA (including cycling) in summer and winter. Both responses were combined to a single variable of leisure-time PA. The PA variable is categorized as (1] “(almost) no activity”, (2) “about 1 hour per week irregularly”, (3) “about 1 hour per week regularly”, and (4) “regularly more than 2 hours per week”. Categories (2) and (3) have been combined in all statistical analyses. The questions about leisure-time PA were derived from the German Cardiovascular Prevention Study conducted between 1979 and 1995. By using a physical activity diary as comparison, these questions have been validated in the KORA population [30].

#### Covariates

Further covariables assessed at all three measurement points and included in the evaluation were sex, age and formal education (primary, secondary, and tertiary education). In the sensitivity analysis history of cancer, myocardial infarction, and stroke (all three coded as binary variables) were introduced as covariates.

### Statistics

Descriptive statistics are reported for all participants, stratified by sex, PA change status, and weight change status.

**Fig 1:**
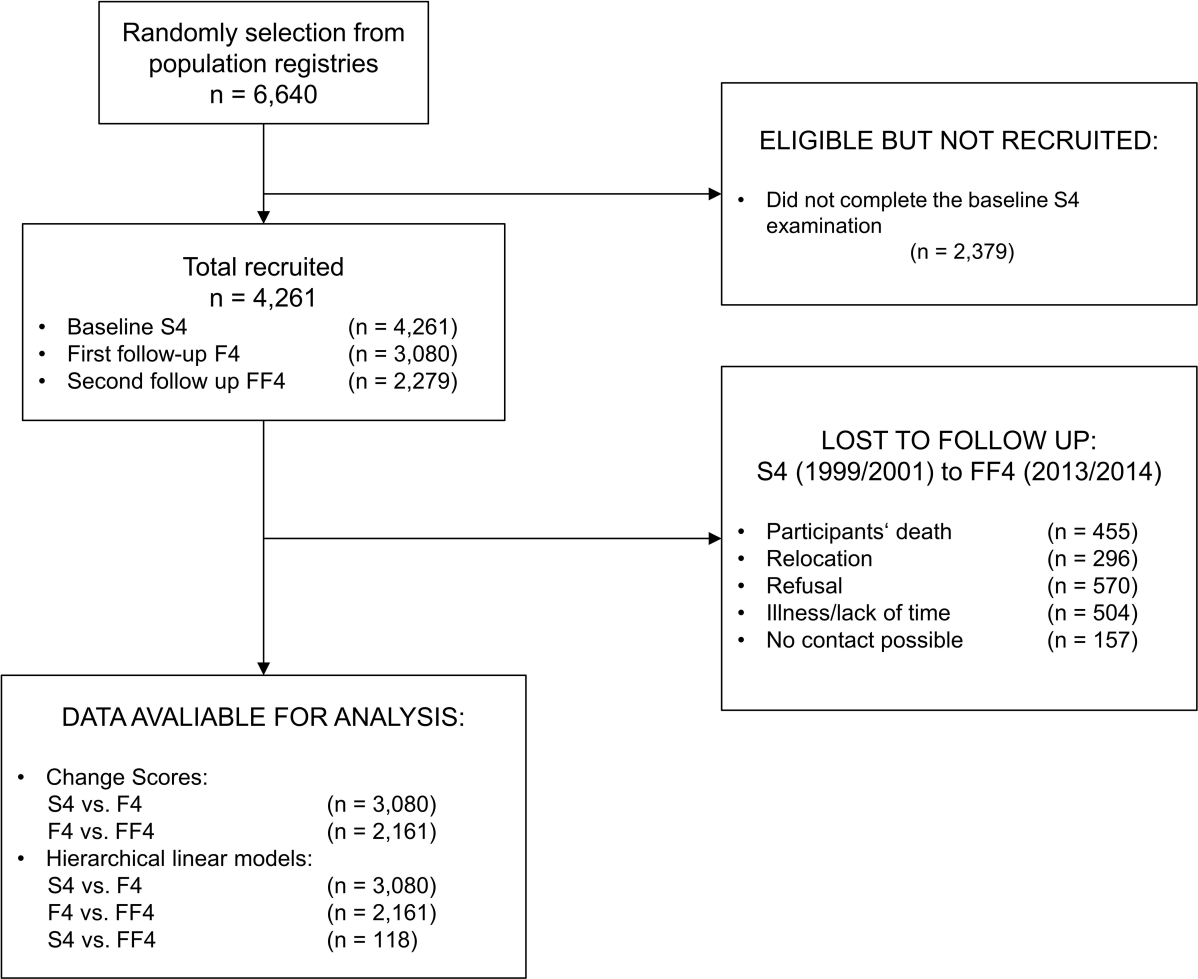
Overview of the study population and the number of cases in the analyses.

For the inference statistical analyses, two distinct statistical approaches have been chosen: change score analyses and hierarchical linear models (HLM). With the aim of investigating HRQL in the general healthy population and in order to prevent confounding, individuals with incident cases of cancer (n = 206), myocardial infarction (n = 52), or stroke (n = 56) as well as pregnant individuals (n = 20) were excluded. Fig 1 gives an overview of how the study size was conceived and how many cases were considered in the analyses.

#### Change score analyses

The first approach, the change score analysis, considers the change between each of the two 7-year follow-up examinations. PA change status is categorized into the categories “decreased PA”, “stable PA”, and “increased PA”, depending on information on F4 compared with S4 and on FF4 compared with F4. A “stable PA” indicates no change in the PA categories between the two comparing measurements. As in previous work [25], stable weight is defined as a maximum deviation of 5% from the baseline weight compared with follow-up. If a person weighs more than 5%/less than 5% of her/his baseline weight, weight change is characterized as increased/decreased. To increase statistical power, we overlaid the two 7-year follow-up periods, i.e., the first period between S4 and F4, which comprises 3,080 participants, and the second period between F4 and FF4, which comprises 2,161 participants. This approach results in a model with 5,241 observations of 3,080 independent participants. To account for the dependence of repeated observations (change from S4 to F4 and from F4 to FF4), the person ID was included in the model as a random intercept.

An ordinary least square regression model was fitted with change in PA/weight as independent variables and change in HRQL as the dependent variable. In the literature, this approach is often referred to as change score analysis [31]. As it is expected that effects of PA/weight change on HRQL depend on baseline PA/weight, we additionally introduced an interaction term in the model between the baseline status and the change variable. PA status is based on the baseline value of PA with (1) = “no/low PA”, (2) = “moderate PA”, and (3) = “high PA”. Weight status is based on the baseline BMI information and presented in the categories “normal weight (BMI ≥18.5–<25 kg/m^2^)”, “overweight (BMI ≥25–<30 kg/m^2^)”, and “obese (BMI ≥30 kg/m^2^)”. The change score models were adjusted for covariates sex, age, and formal education. Statistical significance in change in HRQL for increased or decreased PA/weight was tested against the reference category “stable PA”. The model in use is described through the following equation:

$$\begin{array}{l} \Delta HRQL_{j,i}\\ =\beta_{0}+\beta_{weight\;change,j,i}X_{weight\;change,j,i}\\ +\beta_{weight\;change\;\times \;baseline\;weight,j,i}X_{weight\;change,j,i}X_{baseline\;weight,j,i}\;+\;\beta_{PA\;change,j,i}X_{PA\;change,j,i}\\ +\beta_{PA\;change\times baseline\;PA,j,i}X_{PA\;change,j,i}X_{baseline\;PA,j,i}+\beta_{j,i}X_{j,i}+v_{i}+\varepsilon_{ji} \end{array}$$

in which Δ*HRQL*_*i*,*j*_ is the difference in HRQL between the follow-up and baseline of observation *j* and individual *i*, *β*_*change*,*i*,*j*_ *X*_*change*,*i*,*j*_ is the main effect of the change in weight/PA, *β*_*change* × *baseline*,*i*,*j*_*X*_*change*,*i*,*j*_ × *X*_*baseline weight*,*i*,*j*_ is the interaction effect between baseline weight/PA and change in weight/PA over the follow-up period, *β*_*i*,*j*_*X*_*i*,*j*_ is the linear predictor of covariates, *v*_*i*_ is the random individual-specific deviation from *β*_0_, and *ε*_*i*,*j*_ is the error term.

#### Hierarchical linear modeling

In a second approach, we applied hierarchical linear models. This method overcomes the limitations of other traditional repeated measuring techniques, such as the change score models, and allows the introduction of time-varying covariates. Hedeker [32] proposed a methodological approach to distinguish between between-subject estimates (cross-sectional association) and within-subject estimates (longitudinal association). This two-level approach considers the time points as time nested in individuals and divides the original independent variable into the mean over time (between-subject estimate) and the deviation from the mean over time (within-subject estimate). However, this approach is only applicable to continuous independent variables. Therefore, we could apply this approach for BMI, but not for PA, which was introduced into the model as a regular time-varying covariate. The model in use is described through the following equation:

$${HRQL}_{ji}=\beta_{0}+\beta_{BS}(\bar{{BMI}_{i}})+\beta_{Time}{\left(Time\right)}_{ji}+\beta_{WS}({BMI}_{ji}-\bar{{BMI}_{i}})+\beta_{PA}(PA)+\beta_{j,i}X_{j,i}+v_{0i}+\varepsilon_{ji}$$

in which *HRQL*_*ji*_ is the quality of life value at time point *j* of individual *i*, *β*_0_ is the global intercept, *β*_*Time*_ represents the average change over time, *β*_*BS*_ and *β*_*WS*_ indicate the between-subject and within-subject associations, *β*_*PA*_ is the regression coefficient for the parameter PA and can be interpreted as a general time-varying covariate in the model, *β*_*j*,*i*_*X*_*j*,*i*_ is the linear predictor of other time-varying and time-invariant covariates, *v*_0*i*_ is the random individual-specific deviation from *β*_0_, and *ε*_*ji*_ is the error term, which is assumed to be normally and conditionally independently distributed from *v*_0*i*_ with zero mean and common variance *σ*^2^.

#### Supplementary analyses

For the HLM, additional stratified analyses were carried out. First, to consider possible gender distinctions, HLMs were stratified by sex. Second, to factor in potential floor and ceiling effects in the categorical PA variable, baseline PA was dichotomized with the median as cut-off point. Then, stratified analyses were run, based on this variable. Third, as we assumed that age might influence the results, we decided to perform stratified analyses based on a binary age variable, which was created using the median age. Fourth, in order to see whether participants who had extreme changes in their weight might influence the results, we performed a sensitivity analysis in which we cut off the 5^th^ and 95^th^ percentile of weight change. In a final analysis, we included the previously omitted cases with incident diseases and adjusted the model for these diseases. All results presented in tables in this article are based on the described statistical models. Results of the supplementary analyses are only mentioned in the text when they are significant. Tables with detailed results for the supplementary analyses are presented in the appendix.

All data analyses were carried out with SAS 9.3 (SAS Institute, Cary, NC, USA). The significance level was set at α = 0.05 for all analyses.

## Results

### Participants

In total, 3,080 participants had at least one follow-up examination. Of these, 1,486 (48.25%) were male. Individuals had a mean age of 49.0 years (SD = 13.27) at S4 in 2000. Mean BMI at S4 was 27.1 (SD = 4.6), and 32.6% were of normal weight. Some 66.9% of the cohort were either overweight or already obese. For the first follow-up, 57.9% of individuals remained of stable weight, 11.2% lost and 30.9% gained weight of at least 5% over 7 years. For the comparison between F4 and FF4, 65% remained stable, 13.9% decreased, and 20.8% increased their weight. Regarding the PA status, 31.6% were not or only a little active, almost half of the participants had a moderate (47.9%) PA status, and 20.5% were highly active. On average, 56.0% of the cohort remained stable in their PA behavior. Some 23.4% showed an increase and 20.7% showed a decrease in PA during the first 7-year period. During the second follow-up period, 59.3% of the participants maintained PA, 20.6% increased, and 20.1% decreased their PA behavior. Further descriptive statistics are presented in Table 1. Additional information on PA/weight change can be found in S1 Table.

**Table 1:** Descriptive statistics of the study population at baseline.

	All	Men	Women
**All (%)**	3,080 (100.0)	1,486 (48.3)	1,594 (51.8)
**Men (%)**	1,486 (48.3)		
**Women (%)**	1,594 (51.8)		
**Age, Mean (SD)**	49.0 (13.3)	49.60 (13.4)	48.5 (13.2)
***Education***^***#***^			
**Primary (%)**	1,589 (51.7)	773 (52.1)	816 (51.3)
**Secondary (%)**	757 (24.6)	293 (19.8)	464 (29.2)
**Tertiary (%)**	729 (23.7)	417 (28.1)	312 (19.6)
**BMI, Mean (SD)**	27.1 (4.6)	27.5 (3.9)	26.8 (5.1)
***Weight status***			
**Underweight (%)**	15 (0.5)	2 (0.1)	13 (0.8)
**Normal (%)**	999 (32.6)	366 (24.7)	633 (40.1)
**Overweight (%)**	1,364 (44.6)	796 (53.7)	568 (36.0)
**Obese (%)**	683 (22.3)	318 (21.5)	365 (23.1)
***PA status***^***‡***^			
**Inactive (%)**	968 (31.6)	465 (31.5)	503 (31.6)
**Moderate (%)**	1,470 (47.9)	663 (44.9)	807 (50.8)
**Highly active (%)**	630 (20.5)	350 (23.7)	280 (17.6)
**Pregnancy present (%)**	14 (0.5)		14 (0.9)
**MI any time (%)**	57 (1.9)	48 (3.2)	9 (0.6)
**Stroke any time (%)**	32 (1.0)	19 (1.3)	13 (0.8)
**Cancer any time (%)**	128 (4.2)	52 (3.5)	76 (4.8)

% = column percentage; SD = standard deviation, MI = myocardial infarction

^#^ Education levels refer to the German “Abitur” (primary), “mittlere Reife” (secondary), and “Hauptschule” (tertiary)

^*‡*^ PA status is based on the PA variable categories with [1] = inactive, [2&3] = moderately active, and [4] = highly active

### Change score analyses

**Fig 2:**
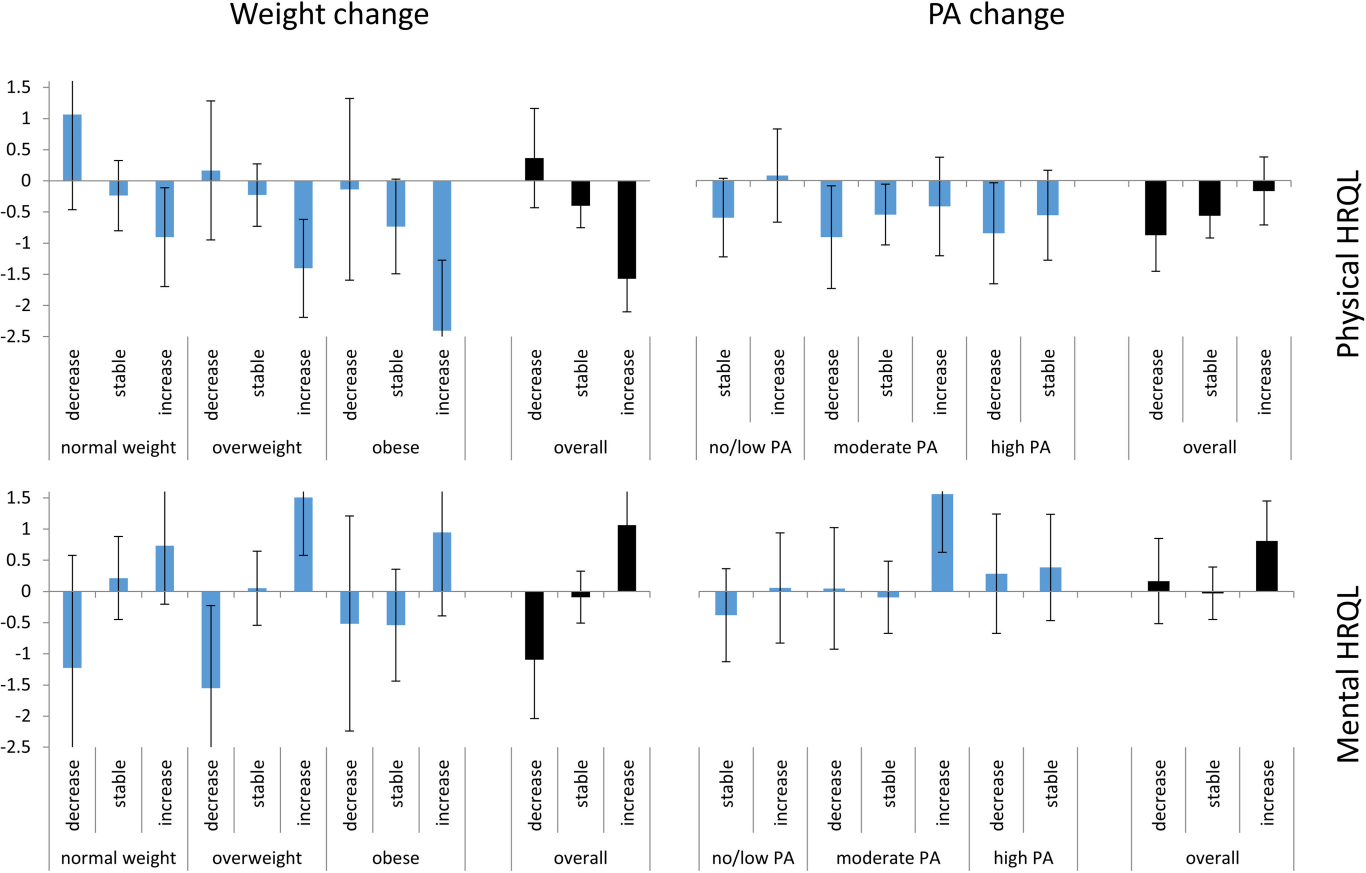
Absolute adjusted mean change scores for PA change and weight change. Abbreviations: HRQL = health-related quality of life, PA = physical activity. Mean change in HRQL for PA change groups and weight change groups. Ordinary least square regression models were adjusted for sex, age, education, and weight/PA change. Error bars indicate 95% confidence intervals. Weight change models are based on 2,643 individuals and 4,193 observations. PA change models are based on 2,659 individuals and 4,218 observations.

The adjusted means in change in PA/weight for the different PA/weight groups are presented in Fig 2. The same figure with supplementary data can be found in the appendix (S1 Fig).

#### Weight change and HRQL

Overall, weight gain led to declines in physical HRQL (overall estimate = –1.57, CI: –2.10 to –1.04, p-value < 0.001). This effect was more pronounced in people with high baseline weight, i.e., overweight or obese people. However, overall, weight gain had a positive influence on mental HRQL (overall estimate = 1.06, CI: 0.44 to 1.69, p-value = 0.003).

#### PA change and HRQL

People who increased their PA from baseline to follow-up had smaller deductions in physical HRQL throughout all PA groups. Nevertheless, these findings never became significant compared with the reference category “stable PA”. Regarding mental HRQL, an increase in PA seems to be related to significantly better results (estimate = 0.81, CI: 0.16 to 1.45, p-value = 0.033). Especially for participants who were moderately active at baseline, an increase in PA resulted in significantly better mental HRQL (estimate = 1.56, CI: 0.62 to 2.49, p-value = 0.003).

### Hierarchical linear models

Results of the HLMs are presented in Table 2. The between-subject effect displays the cross-sectional association, and the within-subject effect reports the longitudinal association of BMI and HRQL. As the PA effects are based on categorical variables, estimates are compared with the reference category “high PA”.

**Table 2:** Results of the HLMs.

	Physical HRQL				Mental HRQL			
Effect	β	95% CI		p-value	β	95% CI		p-value
**BMI (between subjects)**	–0.241	–0.297	–0.186	<0.0001	0.004	–0.057	0.065	0.901
**BMI (within subjects)**	–0.308	–0.433	–0.182	<0.0001	0.384	0.239	0.529	<0.0001
PA (no/low)*	–1.881	–2.417	–1.345	<0.0001	–1.080	–1.690	–0.469	0.001
PA (moderate)*	–0.688	–1.148	–0.229	0.003	–0.870	–1.395	–0.344	0.001

*Compared with reference PA (high); β = parameter estimate; CI = confidence interval

Model based on 2,665 individuals and 6,917 observations.

#### Weight change and HRQL

Looking at the physical domain of HRQL, the BMI between-subject estimate indicates that a higher BMI is associated with impairments in HRQL (β = –0.241, CI: –0.297 to –0.186, p-value = <0.0001). The BMI within-subject estimate shows a decrease in physical HRQL for each increase of one BMI point (β = –0.308, CI: –0.433 to –0.182, p-value = <0.0001). In contrast to the physical scale, associations for the BMI between subjects (β = 0.004, CI: –0.057 to 0.065, p-value = 0.901) are not significant. The estimates for the BMI within-subjects (β = 0.384, CI: 0.239 to 0.529, p-value = <0.0001) show significant results for the mental domain of HRQL.

#### PA and HRQL

“Low PA” (β = –1.881, CI: –2.417 to –1.345, p-value = <0.0001) or “moderate PA” (β = –0.688, CI: –1.148 to –0.229, p-value = 0.003) is associated with significantly lower physical HRQL in comparison with “high PA”. In comparison with “high PA”, “low PA” (β = –1.080, CI: –1.690 to –0.469, p-value = 0.001) and “moderate PA” (β = –0.870, CI: –1.395 to –0.344, p-value = 0.001) indicate a significantly poorer mental HRQL. However, as PA is included in the model as a simple time-varying covariate, the effect estimates rather describe the cross-sectional relationship between PA and HRQL.

### Supplementary analyses

In supplementary analyses, which were adjusted for the diseases cancer, myocardial infarction, and stroke, no major changes in the estimates were observed (S2 Table). Analysis in which we cut off the 5^th^ and 95^th^ percentile of weight loss showed no change compared to the estimates of the main analysis (S3 Table). Stratified analyses for male and female participants showed a significantly smaller BMI within-subject estimate for women on the physical domain of HRQL (S4 Table). Females who were “less active” had lower mental HRQL scores than males as well (S5 Table). In models stratified for median age at baseline, older participants who were “less active” had significantly lower PA estimates on the physical HRQL scale than younger participants (S4 Table). On the mental scale, older people had significantly better BMI within-subject estimates than younger participants (S5 Table). Models stratified for median PA at baseline showed no significant estimates for all parameters (S4 and S5 Tables). Results of the supplementary analyses are presented in detail in the appendix.

## Discussion

In the present study, the longitudinal associations of PA change and weight change with HRQL have been investigated using change score analyses and HLM. We analyzed data from a population-based cohort including three measurement points, S4 (2000), F4 (2007), and FF4 (2014). This cohort study further clarifies the relationship between change in PA/weight and HRQL. A change to a physically more active lifestyle is positively associated with physical and mental HRQL. Although weight gain is associated with impairments in physical HRQL, there seems to be an inverse relationship between weight gain and mental HRQL. Our analyses showed a constant pattern in which weight gain is associated with higher mental HRQL. Despite the fact that we chose two different methodological approaches to investigate the association between PA/weight change and HRQL, the results are homogeneous. In both the change score analyses and the hierarchical linear models, the effects for the association are aligned. However, comparing the between-subject and the within-subject estimates of the hierarchical linear models, the effects for the physical HRQL domain do not differ substantially as their confidence intervals are overlapping. Only for the mental domain do the two estimates differ. From a methodological perspective, results from a longitudinal study are less prone to selection or recall bias.

### Comparison with findings from other studies

Previous longitudinal research in this field has reported a positive relationship between PA change and HRQL [10–12, 22, 23]. Our results describe the same positive association and are therefore in line with previous findings. In line with other studies [12, 22], we found significant differences for males and females in some of the supplementary analyses. As HRQL was assessed with the SF-12, we can only distinguish between a mental and a physical component summary of HRQL. Therefore, we cannot provide statements on further subdomains of HRQL as other studies have done [12, 22].

When it comes to previous longitudinal research on weight change and HRQL, our findings on physical HRQL are consistent with other studies. Numerous studies describe an inverse relationship between weight gain and physical HRQL [14–16, 21, 24–26]. Our data imply improved mental HRQL for weight gainers. Although this is not the case in all previous studies [14], there are several other studies that support this result [21, 25, 26]. Inconsistencies with the results from Döring et al. [14] might eventuate in the fact, that two different questionnaires on HRQL were utilized. Compared with the study by Laxy et al., which analyzed the relationship between weight and HRQL using the same data over a 7-year time period, this work comprised a new measurement point and therefore considers an additional 7 years of observation. Furthermore, we extended the research question and added the association between PA change and HRQL. Our findings are generally consistent with the results of Laxy and colleagues.

### Clinical relevance

In the literature, clinically relevant thresholds for HRQL change assessed via SF-12 have been established. Warkentin et al. report a change from three to five points on the SF-12 scale as a minimal clinically important difference [33]. Our estimates do not surpass this threshold, unless we consider an enormous change in BMI of more than ten points. Given the fact that our cohort showed a mean change in weight of 1.8 kg from 2000 to 2007 and 0.8 kg from 2007 to 2014, we cannot report clinical relevance for our results. However, it is well known that HRQL typically declines over age. Taking the results from Ellert et al. [7] as a representative standard, we can compare our results with the natural evolution of HRQL through aging. Over a timespan of ten years (age group 40–49 until 50–59), the data from Ellert et al. show a decline of two points for the physical component of HRQL and an increase of .3 points for the mental component. Comparing this for example with our physical BMI within-subject estimate (-0.308), a gain of about 6.5 BMI points would resemble to a 10 year decline of HRQL due to aging.

### Limitations

There are several limitations to our study. First, as HRQL can be seen as a holistic concept, which tries to consider all aspects of health, it is only reasonable that it can be influenced by many factors. Although we adjusted our models for several variables, the chances are high that the change in HRQL might have been influenced by a factor we did not adjust for. We tried to counteract this problem by the introduction of the use of longitudinal analyses. Change scores and the within-subject estimate of the HLM compare the same people and, therefore, theoretically can only be confounded by time-varying factors. However, time-varying factors we did not control for might still have biased the effect estimates. Another common problem with observational studies is confounding by indication and reverse causation. As we have no information on participants’ intention to change their PA/weight, we could not differentiate between voluntary and non-voluntary PA/weight change. Another limitation that has to be addressed is the manner in which PA was assessed in the study. PA is a self-reported variable, which was gathered in standardized interviews, and therefore recall bias or measurement errors are likely to have occurred. Moreover, PA is depicted as a categorical variable, which precludes PA from the more sophisticated methodological approaches used in the analyses focusing on weight. Beyond that, the considerably reduced size of the cohort through the 14 years of observation might result in biased effects, as healthier and more active people are more likely to remain in the cohort. Nevertheless, the fact that the between-subject and within-subject estimates are similar in most cases indicates that this potential bias might not be that substantial. Although the combination of two methodological approaches lead to a certain robustness of our results, it has to be mentioned that multiple statistical models generate alpha risk inflation.

### Strengths

The strength of our study is its longitudinal design. We were able to investigate a population-based cohort over 14 years including three measurement points. Additionally, all anthropometric information is based on objectively measured data. The inclusion of PA as a lifestyle factor is another advantage of our study, which has been noted as an important factor to consider in other publications [14]. A major strength of the study is the application of two methodological approaches. With the multi-level approach and the inclusion of between-subject and within-subject estimates, we could report and compare cross-sectional and longitudinal associations.

### Conclusion

In conclusion, this study further contributes to the so far rather sparse evidence concerning longitudinal associations on PA change/weight change and HRQL. Our results stress the importance of interventions on PA and weight. Nonetheless, further research in this field is needed to reveal a causal relationship between PA/weight change and HRQL. Concerning PA, objective assessment of PA patterns through accelerometers or wearable devices is indispensable to infer precise and reliable effect estimates.

## Supporting information

S1 Fig Absolute adjusted mean change scores for PA change and weight change. (TIF)

S1 Table Additional descriptive statistics for the change groups. (DOCX)

S2 Table Results of HLM with cases included that were excluded from the basic model. (DOCX)

S3 Table Results of the HLM without 5th and 95th percentile of weight loss. (DOCX)

S4 Table Results of stratified analyses of the basic model for physical HRQL. (DOCX)

S5 Table Results of stratified analyses of the basic model for mental HRQL. (DOCX)
