# Supplementary material for: The longitudinal association between change in physical activity, weight, and health-related quality of life: Results from the population-based KORA S4/F4/FF4 cohort study
Source: PLoS One. 2017 Sep 27;12(9):e0185205. doi: 10.1371/journal.pone.0185205 (PMC5617179; doi:10.1371/journal.pone.0185205)
Supplement: S1 Table — (DOCX) [file pone.0185205.s002.docx]

S1 Table. Additional descriptive statistics for the change groups.

|  | **Study** | **Weight change** | | |  | **PA change** | | |
| --- | --- | --- | --- | --- | --- | --- | --- | --- |
|  |  | **Decrease** | **Stable** | **Increase** |  | **Decrease** | **Stable** | **Increase** |
| **All (%)** | S4 vs F4 | 341 (11.2) | 1,765 (57.9) | 940 (30.9) |  | 633 (20.7) | 1,714 (56.0) | 716 (23.4) |
|  | F4 vs FF4 | 300 (13.9) | 1,405 (65.3) | 447 (20.8) |  | 434 (20.1) | 1,281 (59.3) | 446 (20.6) |
| **Men (%)** | S4 vs F4 | 148 (10.0) | 887 (60.1) | 442 (29.9) |  | 316 (21.5) | 831 (56.4) | 326 (22.1) |
|  | F4 vs FF4 | 140 (13.4) | 718 (68.6) | 189 (18.1) |  | 211 (20.1) | 633 (60.3) | 206 (19.6) |
| **Women (%)** | S4 vs F4 | 193 (12.3) | 878 (56.0) | 498 (31.7) |  | 317 (19.9) | 883 (55.5) | 390 (24.5) |
|  | F4 vs FF4 | 160 (14.5) | 687 (62.2) | 258 (23.4) |  | 223 (20.1) | 648 (58.3) | 240 (21.6) |
| **Age, Mean (SD)*** | S4 vs F4 | 52.5 (13.9) | 50.6 (13.0) | 44.9 (14.4) |  | 48.2 (14.3) | 49.4 (13.1) | 48.6 (12.6) |
|  | F4 vs FF4 | 59.3 (12.7) | 54.6 (12.2) | 48.7 (10.3) |  | 54.7 (12.8) | 54.3 (12.2) | 52.6 (11.9) |
| **Education** |  |  |  |  |  |  |  |  |
| **Primary (%)** | S4 vs F4 | 196 (12.4) | 931 (59.0) | 451 (28.6) |  | 317 (20.1) | 883 (55.9) | 380 (24.1) |
|  | F4 vs FF4 | 168 (15.9) | 677 (64.1) | 212 (20.1) |  | 228 (21.5) | 631 (59.6) | 200 (18.9) |
| **Secondary (%)** | S4 vs F4 | 75 (10.1) | 404 (54.2) | 267 (35.8) |  | 157 (20.8) | 408 (54.1) | 189 (25.1) |
|  | F4 vs FF4 | 66 (12.1) | 365 (67.1) | 113 (20.8) |  | 117 (21.4) | 320 (58.5) | 110 (20.1) |
| **Tertiary (%)** | S4 vs F4 | 70 (9.8) | 426 (59.4) | 221 (30.8) |  | 159 (21.8) | 423 (58.1) | 146 (20.1) |
|  | F4 vs FF4 | 65 (11.9) | 360 (65.9) | 121 (22.2) |  | 89 (16.2) | 327 (59.5) | 134 (24.4) |
| **BMI, Mean (SD)** | S4 vs F4 | 28.7 (5.2) | 27.1 (4.4) | 26.6 (4.5) |  | 27.1 (4.7) | 27.1 (4.5) | 27.2 (4.6) |
|  | F4 vs FF4 | 28.8 (4.8) | 27.1 (4.6) | 27.1 (4.7) |  | 27.6 (4.8) | 27.3 (4.7) | 27.0 (4.7) |
| **Weight status** |  |  |  |  |  |  |  |  |
| **Underweight (%)** | S4 vs F4 | 0 (0.0) | 7 (46.7) | 8 (53.3) |  | 2 (13.3) | 9 (60.0) | 4 (26.7) |
|  | F4 vs FF4 | 0 (0.0) | 7 (77.8) | 2 (22.2) |  | 2 (22.2) | 5 (55.6) | 2 (22.2) |
| **Normal (%)** | S4 vs F4 | 77 (7.7) | 559 (56.1) | 361 (36.2) |  | 219 (22.03) | 551 (55.4) | 224 (22.5) |
|  | F4 vs FF4 | 66 (9.2) | 484 (67.3) | 169 (23.5) |  | 140 (19.5) | 417 (58.0) | 162 (22.5) |
| **Overweight (%)** | S4 vs F4 | 166 (12.3) | 811 (59.9) | 378 (27.9) |  | 265 (19.5) | 766 (56.5) | 325 (24.0) |
|  | F4 vs FF4 | 129 (14.2) | 607 (66.9) | 172 (18.9) |  | 177 (19.5) | 557 (61.3) | 174 (19.2) |
| **Obese (%)** | S4 vs F4 | 98 (14.4) | 388 (57.1) | 193 (28.4) |  | 141 (20.8) | 379 (55.8) | 159 (23.4) |
|  | F4 vs FF4 | 105 (20.4) | 307 (59.5) | 104 (20.2) |  | 112 (21.7)) | 299 (58.0) | 105 (20.4) |
| **PA status** |  |  |  |  |  |  |  |  |
| **Inactive (%)** | S4 vs F4 | 138 (14.5) | 513 (53.8) | 303 (31.8) |  | 0 (0.0) | 574 (59.5) | 390 (40.5) |
|  | F4 vs FF4 | 106 (17.5) | 375 (61.8) | 126 (20.8) |  | 0 (0.0) | 372 (60.7) | 241 (39.3) |
| **Moderate (%)** | S4 vs F4 | 144 (9.9) | 870 (59.8) | 442 (30.4) |  | 342 (23.3) | 801 (54.5) | 326 (22.2) |
|  | F4 vs FF4 | 135 (13.7) | 638 (64.9) | 210 (21.4) |  | 188 (19.1) | 592 (60.1) | 205 (20.8) |
| **Highly active (%)** | S4 vs F4 | 59 (9.5) | 375 (60.1) | 190 (30.5) |  | 291 (46.2) | 339 (53.8) | 0 (0.0) |
|  | F4 vs FF4 | 59 (10.5) | 392 (69.8) | 111 (19.8) |  | 246 (43.7) | 317 (56.3) | 0 (0.0) |
| **Pregnancy present (%)** | S4 vs F4 | 0 (0.0) | 1 (50.0) | 1 (50.0) |  | 6 (42.9) | 5 (35.7) | 3 (21.4) |
|  | F4 vs FF4 | 0 (0.0) | 1 (100.0) | 0 (0.0) |  | 3 (50.0) | 0 (0.0) | 3 (50.0) |
| **MI any time (%)** | S4 vs F4 | 8 (14.3) | 31 (55.4) | 17 (30.4) |  | 16 (28.1) | 35 (61.4) | 6 (10.5) |
|  | F4 vs FF4 | 16 (31.4) | 27 (52.9) | 8 (15.7) |  | 10 (18.9) | 27 (50.9) | 16 (30.2) |
| **Stroke any time (%)** | S4 vs F4 | 4 (14.3) | 18 (64.3) | 6 (21.4) |  | 9 (29.0) | 16 (51.6) | 6 (19.4) |
|  | F4 vs FF4 | 4 (20.0) | 12 (60.0) | 4 (20.0) |  | 5 (22.7) | 13 (59.1) | 4 (18.2) |
| **Cancer any time (%)** | S4 vs F4 | 16 (12.7) | 79 (62.7) | 31 (24.6) |  | 31 (24.2) | 63 (49.2) | 34 (26.6) |
|  | F4 vs FF4 | 25 (16.8) | 102 (68.5) | 22 (14.8) |  | 36 (24.2) | 81 (54.4) | 32 (21.5) |

% = row percentage; SD = standard deviation; MI = myocardial infarction
