# Supplementary material for: The longitudinal association between change in physical activity, weight, and health-related quality of life: Results from the population-based KORA S4/F4/FF4 cohort study
Source: PLoS One. 2017 Sep 27;12(9):e0185205. doi: 10.1371/journal.pone.0185205 (PMC5617179; doi:10.1371/journal.pone.0185205)
Supplement: S2 Table — (DOCX) [file pone.0185205.s003.docx]

S2 Table. Results of HLM with cases included that were excluded from the basic model.

|  | **Physical HRQL** | | | |  | **Mental HRQL** | | | |
| --- | --- | --- | --- | --- | --- | --- | --- | --- | --- |
| **Effect** | **β** | **95% CI** | | **p-value** |  | **β** | **95% CI** | | **p-value** |
| **BMI (between subjects)** | –0.253 | –0.306 | –0.201 | <0.0001 |  | 0.006 | –0.052 | 0.063 | 0.850 |
| **BMI (within subjects)** | –0.285 | –0.405 | –0.166 | <0.0001 |  | 0.383 | 0.246 | 0.520 | <0.0001 |
| **PA (no/low)*** | –2.040 | –2.547 | –1.533 | <0.0001 |  | –1.262 | –1.834 | –0.689 | <0.0001 |
| **PA (moderate)*** | –0.746 | –1.184 | –0.307 | 0.001 |  | –0.861 | –1.358 | –0.365 | 0.001 |

*Compared with reference PA (high); β = parameter estimate; CI = confidence interval
