# Supplementary material for: The longitudinal association between change in physical activity, weight, and health-related quality of life: Results from the population-based KORA S4/F4/FF4 cohort study
Source: PLoS One. 2017 Sep 27;12(9):e0185205. doi: 10.1371/journal.pone.0185205 (PMC5617179; doi:10.1371/journal.pone.0185205)
Supplement: S3 Table — (DOCX) [file pone.0185205.s004.docx]

S3 Table. Results of the HLM without 5th and 95th percentile of weight loss.

|  | **Physical HRQL** | | | |  | **Mental HRQL** | | | |
| --- | --- | --- | --- | --- | --- | --- | --- | --- | --- |
| **Effect** | **β** | **95% CI** | | **p-value** |  | **β** | **95% CI** | | **p-value** |
| **BMI (between subjects)** | -0.215 | -0.275 | -0.156 | <0.0001 |  | 0.030 | -0.035 | 0.096 | 0.363 |
| **BMI (within subjects)** | -0.308 | -0.513 | -0.103 | 0.003 |  | 0.581 | 0.346 | 0.816 | <0.0001 |
| **PA (no/low)*** | -1.834 | -2.391 | -1.277 | <0.0001 |  | -1.233 | -1.861 | -0.605 | <0.0001 |
| **PA (moderate)*** | -0.715 | -1.190 | -0.240 | 0.003 |  | -0.944 | -1.483 | -0.405 | 0.001 |

*Compared with reference PA (high); β = parameter estimate; CI = confidence interval
