# Supplementary material for: The longitudinal association between change in physical activity, weight, and health-related quality of life: Results from the population-based KORA S4/F4/FF4 cohort study
Source: PLoS One. 2017 Sep 27;12(9):e0185205. doi: 10.1371/journal.pone.0185205 (PMC5617179; doi:10.1371/journal.pone.0185205)
Supplement: S4 Table — (DOCX) [file pone.0185205.s005.docx]

S4 Table. Results of stratified analyses of the basic model for physical HRQL.

| **Physical HRQL** | | | | |
| --- | --- | --- | --- | --- |
| **Effect** | **β** | **95% CI** | | **p-value** |
| ***Basic model stratified by sex*** | | | | |
| **Male BMI (between subjects)** | –0.220 | –0.308 | –0.132 | 0.527 |
| **Female BMI (between subjects)** | –0.254 | –0.326 | –0.182 |  |
| **Male BMI (within subjects)** | –0.146 | –0.336 | 0.044 | 0.034 |
| **Female BMI (within subjects)** | –0.415 | –0.582 | –0.249 |  |
| **Male PA (no/low)** | –2.306 | –3.043 | –1.568 | 0.338 |
| **Female PA (no/low)** | –1.501 | –2.279 | –0.724 |  |
| **Male PA (moderate)** | –0.792 | –1.430 | –0.154 |  |
| **Female PA (moderate)** | –0.568 | –1.227 | 0.091 |  |
| ***Basic model stratified by median age (years)*** | | | | |
| **Age ≤46 BMI (between subjects)** | –0.214 | –0.282 | –0.145 | 0.206 |
| **Age >46 BMI (between subjects)** | –0.266 | –0.351 | –0.180 |  |
| **Age ≤46 BMI (within subjects)** | –0.312 | –0.469 | –0.156 | 0.109 |
| **Age >46 BMI (within subjects)** | –0.362 | –0.562 | –0.161 |  |
| **Age ≤46 PA (no/low)** | –1.159 | –1.838 | –0.479 | 0.005 |
| **Age >46 PA (no/low)** | –2.713 | –3.542 | –1.884 |  |
| **Age ≤46 PA (moderate)** | –0.089 | –0.648 | 0.470 |  |
| **Age >46 PA (moderate)** | –1.440 | –2.176 | –0.703 |  |
| ***Basic model stratified by median PA*** | | | | |
| **PA (under median) BMI (between subjects)** | –0.209 | –0.287 | –0.132 | 0.709 |
| **PA (above median) BMI (between subjects)** | –0.265 | –0.345 | –0.185 |  |
| **PA (under median) BMI (within subjects)** | –0.209 | –0.384 | –0.033 | 0.366 |
| **PA (above median) BMI (within subjects)** | –0.417 | –0.597 | –0.237 |  |
| **PA (under median) PA (no/low)** | –2.061 | –3.172 | –0.949 | 0.215 |
| **PA (above median) PA (no/low)** | –2.218 | –3.140 | –1.297 |  |
| **PA (under median) PA (moderate)** | –1.296 | –2.387 | –0.205 |  |
| **PA (above median) PA (moderate)** | –0.382 | –0.886 | 0.122 |  |

β = parameter estimate; CI = confidence interval; p-values result from an interaction term between sex/median age/median PA and the parameters listed
