# Supplementary material for: The longitudinal association between change in physical activity, weight, and health-related quality of life: Results from the population-based KORA S4/F4/FF4 cohort study
Source: PLoS One. 2017 Sep 27;12(9):e0185205. doi: 10.1371/journal.pone.0185205 (PMC5617179; doi:10.1371/journal.pone.0185205)
Supplement: S5 Table — (DOCX) [file pone.0185205.s006.docx]

S5 Table. Results of stratified analyses of the basic model for mental HRQL.

| **Mental HRQL** | | | | |
| --- | --- | --- | --- | --- |
| **Effect** | **β** | **95% CI** | | **p-value** |
| ***Basic model stratified by sex*** | | | | |
| **Male BMI (between subjects)** | –0.060 | –0.155 | 0.034 | 0.269 |
| **Female BMI (between subjects)** | 0.053 | –0.029 | 0.135 |  |
| **Male BMI (within subjects)** | 0.293 | 0.082 | 0.503 | 0.190 |
| **Female BMI (within subjects)** | 0.445 | 0.245 | 0.645 |  |
| **Male PA (no/low)** | –0.444 | –1.250 | 0.361 | 0.048 |
| **Female PA (no/low)** | –1.732 | –2.647 | –0.817 |  |
| **Male PA (moderate)** | –0.590 | –1.289 | 0.109 |  |
| **Female PA (moderate)** | –1.174 | –1.955 | –0.394 |  |
| ***Basic model stratified by median age (years)*** | | | | |
| **Age ≤46 BMI (between subjects)** | 0.008 | –0.078 | 0.094 | 0.992 |
| **Age >46 BMI (between subjects)** | 0.006 | –0.082 | 0.094 |  |
| **Age ≤46 BMI (within subjects)** | 0.213 | 0.023 | 0.404 | 0.023 |
| **Age >46 BMI (within subjects)** | 0.591 | 0.366 | 0.815 |  |
| **Age ≤46 PA (no/low)** | –0.705 | –1.543 | 0.132 | 0.703 |
| **Age >46 PA (no/low)** | –1.469 | –2.366 | –0.572 |  |
| **Age ≤46 PA (moderate)** | –0.672 | –1.359 | 0.015 |  |
| **Age >46 PA (moderate)** | –1.096 | –1.900 | –0.292 |  |
| ***Basic model stratified by median PA*** | | | | |
| **PA (under median) BMI (between subjects)** | 0.039 | –0.046 | 0.124 | 0.151 |
| **PA (above median) BMI (between subjects)** | –0.032 | –0.122 | 0.058 |  |
| **PA (under median) BMI (within subjects)** | 0.355 | 0.155 | 0.555 | 0.386 |
| **PA (above median) BMI (within subjects)** | 0.428 | 0.215 | 0.640 |  |
| **PA (under median) PA (no/low)** | –0.690 | –1.944 | 0.564 | 0.076 |
| **PA (above median) PA (no/low)** | –2.030 | –3.105 | –0.955 |  |
| **PA (under median) PA (moderate)** | –0.740 | –1.973 | 0.493 |  |
| **PA (above median) PA (moderate)** | –0.781 | –1.369 | –0.194 |  |

β = parameter estimate; CI = confidence interval; p-values result from an interaction term between sex/median age/median PA and the parameters listed
